# Spatial distribution and the prevalence of speech disorders in the provinces of Iran 

**Published:** 2016

**Authors:** I Hurjui, S Pete, I Bostan

**Affiliations:** * Doctoral School of Economics, “Stefan cel Mare” University, Suceava, Romania; * Babeş–Bolyai” University, Faculty of Economics and Business Administration, Cluj Napoca, Cluj, Romania

**Keywords:** external audit, funding professional integration, labor market, persons with disabilities (PWD)

## Abstract

The paper reveals the presence of certain factors that generate inefficiency - in the area of government programmes for labor market inclusion of persons with disabilities (PWD) -, as outlined by the specific instruments of the (external) public performance audit. The identified causes are mostly related to the fact that the drawing up of the necessary budgets for projects related to the social and professional integration of persons with disabilities was not based on the local needs and, thus, the available material, financial and human resources have been overlooked. Referring to a period of five years (2006-2010), it was pointed out that the methodologies related to the targeted projects did not mention any clear regulations regarding the criteria that have to be met by the applicants participating in the selection for projects funded from non-refundable resources. Subsequently, certain non-refundable contracts have been assigned to certain executors (NGO types), even though they did not meet the criteria either in terms of eligibility or in terms of the completion of the objectives mentioned in the project proposals, therefore resulting in illegal payments made towards such parties.

## Introduction

On December 31st 2013 [**9**,**12**], the total number of persons with disabilities (PWD) in Romania amounted to 709,216 individuals, 97.6% (692,093 persons) of these being under the care of their families and/ or living independently (non-institutionalized) and 2.4% (17,123 persons) being in social security public institutions housing adults with disabilities. According to the UN Convention on the rights of persons with disabilities [**9**], the said persons (PWD) are defined as human individuals with long-term physical, mental, intellectual, or sensory impairments, who, in interaction with various barriers, may hinder their full and effective participation in society on an equal basis with others. The economic decline had a negative impact on the conditions of PWD, both at a European and a national level, thus giving rise to a very stringent need to take action – through special programmes with adequate funding [**3**,**6**,**7**]. What we have tried to reveal through this study is the extent to which we can identify the weaknesses of the implementation/ development of these programmes at an institutional level in Romania, by using the specific instruments of performance audit, and by seeing whether we could generate certain ideas/ opinions, conclusions/ suggestions meant to lead to a visible improvement in the field approached by the present research.

## Material and methods

Our investigation mostly focused on the period following Romania’s immediate accession to the EU, with a particular focus on the 2006-2010 time interval that was marked by certain specific difficulties, in light of the need to apply the EU regulations. The methods used were the interpretation methods, crucial for an updated grasp of the legislation, from both the lawmaker’s and the researcher’s perspective [**5**] and the comparative method, extremely effective in the study and development of the legislation [**4**]. The data and the information used is available to the public, and their validity was guaranteed by their quotation in various papers that were further explicitly quoted, published under the aegis of prestigious organizations/ institutions, such as the Romanian Government, the Romanian Court of Audit (RCA), etc., correlated with the information included in the bulletins of the National Institute of Statistics. The analyses have taken into account the latest editions of the journals published by the above mentioned organizations, more specifically statistical papers, standards, procedural guidebooks, reports [**1**,**11**,**12**,**13**,**14**,**15**]. Actually, the type of social programmes we made reference to were periodically subject to audit procedures by the RCA, as the Supreme Audit Institution, the activities related to these programmes being subjected to performance audit [**2**] (the related reports and summaries were posted on the website in due time). The audit procedures we referred to were conducted only in compliance with the audit standards and the best international practices in the field. Clearly, the audit was conducted by using specific procedures for the examination of financial and non-financial documents, interviews with various people who have specific responsibilities in the field, questionnaires and observations on the actual implementation of the measures taken in order to enhance performance in the development of the programmes that were closely analyzed.

## Results and discussion

**Fig. 1:**
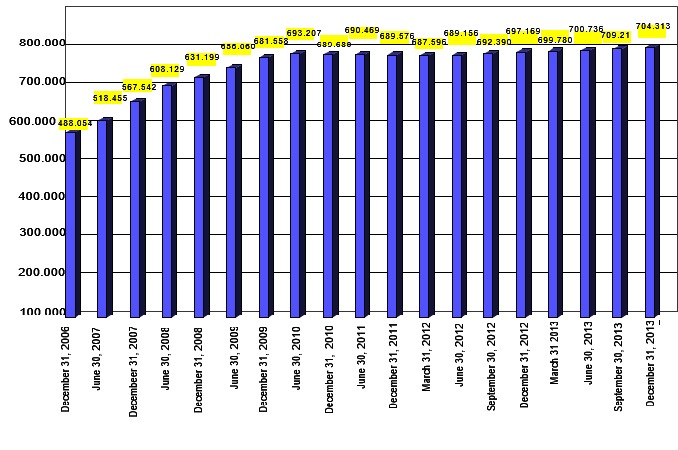
**Fig. 1**Evolution of the number of PWD (December 2006 – December 2013) [**12**]

**Fig. 2:**
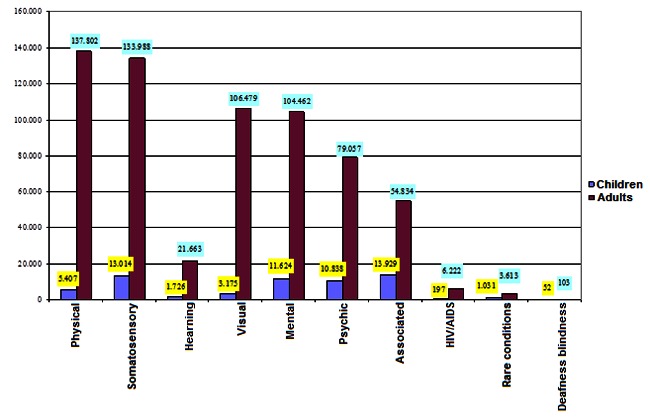
**Fig. 2**Number of PWD, per age and sex groups (December 2013) [**12**]

The total number of PWD in Romania [**12**] amounted to 709,216 persons and we believed that the evolution of these numbers was relatively stable (**Fig. 1,2**). The employment rate of persons with disabilities was significantly lower as compared to the general population [**16**], which was why we further insisted upon the subject of the social programmes for the professional integration of PWD, resorting to the instruments of performance audit, as agreed upon through the official documents of the Romanian Court of Audit [**14**], under the standards and regulations that govern public external audit procedures [**13**]. Moreover [**9**], audit procedures are directed towards identifying the areas that have had a low completion level and pinpoint the possible causes that have led to the failure of completing the established objectives.

After summarizing, analyzing and interpreting the audit samples presented by the National Authority for Persons with Disabilities (NAPD) and National Agency for Employment (NAE) (2006-2010), we have found the following [**14**]:

▯ The legislative factor was supportive through the protective measures taken, as employers have the obligation to take in PWD, but also have the possibility to choose to either pay contributions or purchase products or services resulted from the activity performed by those persons; 

▯ With strict reference to the projects under consideration, only 31 projects with a total value of € 1,022,040, out of a total of 119 such projects with a value amounting to € 7,579,301, have actually focused on increasing the employment of PWD; 

▯ The introduction of the obligation to pay the contributions in cash only in 2009, was imposed to the non-governmental organizations (NGO) that have submitted the project allocation documentation, and has led to a decline in the number of projects implemented in the field under consideration; 

▯ As far as the contract allocation was concerned, there was no clear mention of the obligation of legal persons who competed in the selection of projects funded with non-refundable public funds, to develop actual activities in the field of PWD protection; 

▯ When the projects were implemented, there were cases when the executors have demanded the payment without providing the legal documents to certify that the performed activities met the eligibility criteria/ regulations, thus leading to the occurrence of illegal payments from public funds; 

▯ As far as the monitoring of the projects throughout the period under analysis was concerned, the audit revealed that there were cases when there were no monitoring visits during the post-implementation period, when the funding beneficiary had the obligation to ensure the project sustainability. Moreover, there were no unitary, measurable, and mandatory indicators provided, that would account for the efficiency and effectiveness of the projects accepted for funding from non-refundable public funds. No clear requirements have been expressed in relation to: reporting deadlines, project beneficiary responsibilities, penalties, etc., for those situations in which the project sustainability after implementation was not ensured; 

▯ According to the funding project proposals, the development of such projects directed at the social and professional integration of PWD would have included a number of 1,273 PWD as direct beneficiaries. However, in 2009, the monitoring conducted revealed that the number of beneficiaries amounted to only 563 persons, that was 44.2% of the initial proposals. The number of people employed at the end of 2010 amounted to only 25 persons, even though a number of 31 projects were being implemented during the period under analysis. At NAE, they did not conduct periodic analyses on the need and opportunity to maintain certain types of measures, depending on the evolution and the demands of the labor market; the rate of unemployment not being calculated for PWD, and no data being available on the active population from persons with disabilities. 

Additionally, there was no separate account of the expenses made for the integration on the labor market of PWD, in relation to the adopted measures, in order to ascertain the financial effort of the institution made with the employment of the persons belonging to the category under discussion.

**Fig. 3:**
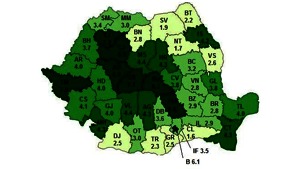
**Fig. 3**Ratio of employed PWD to the total number of persons with disabilities, according to county (%) [**10**]

All these accounted for the low ratio of employed PWD to the total number of persons with disabilities throughout all the countries of Romania (**Fig. 3**).

In order to get a clear picture of the number of people in question, employed in each development region of Romania, with a specific mention of the type of disability, a series of significant data was collected in **Table 1** [**12**]. 

**Table 1:**
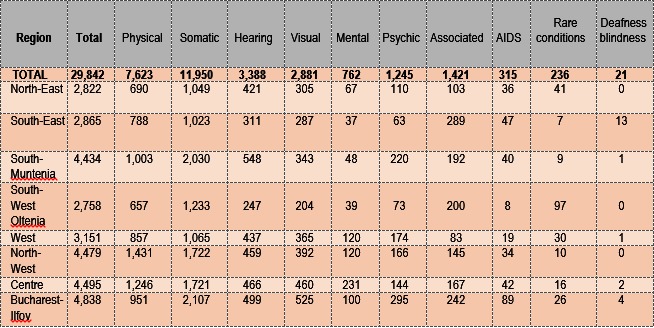
**Table 1**. Disabled persons employed according to the type of deficiency, according to regions (December, 2013) [**12**]

### Measures to be taken in taken in the future

Starting from the information mentioned above, the key objective “access to the labor market of an increasing number of PWD, especially on the free labor market” has been established in a new strategic document [**8**]. 

This was accompanied by the following specific objectives: ♦ the development of the regulatory framework related to the promotion and safeguarding of the rights and freedoms related to the employment of PWD; 

♦ the provision of support for employment, promotion and continuous employment of PWD who are able to work, under social equity conditions: services related to the recovery and maintenance of health, assessment of their ability and work skills, counseling and professional guidance, support for professional training and vocational guidance, support for finding and maintaining a job (assisted employment), specialized transport to/ from the workplace, etc., and workplace adjustment; 

♦ the inclusion in the employment assistance system of all categories of PWD that are able to work, with a particular focus on the most vulnerable groups; 

♦ the stimulation of PWD employment opportunities by directing special protection funds from the passive area towards facilities and social support services aimed at professional training, employment and continuous employment; 

♦ the stimulation and support of employers for hiring PWD; 

♦ the diversification of the vocational training opportunities for PWD; 

♦ the development of specific services providing information and professional guidance and counseling for PWD, particularly for children and young people with disabilities, informing them on the availability of the European resources for employment; 

♦ the improvement of the monitoring mechanisms that measure the impact of the social measures taken to support the employment of PWD. 

## Conclusions

Even though it is difficult to draw conclusions on the efficiency and effectiveness of projects of the kind – as the issued secondary legal provisions do not define the performance indicators needed for project evaluation – we can still mention a few conclusive (critical) elements. Among these, the methodologies related to the targeted projects did not mention clear regulations regarding the criteria that have to be met by the applicants participating in the selection for projects funded from non-refundable resources. Subsequently, certain non-refundable contracts have been assigned to certain executors (NGO types), even though they did not meet the criteria either in terms of eligibility or in terms of the completion of the objectives mentioned in the project proposals, therefore resulting in illegal payments made towards such parties. Moreover, monitoring visits during the period following the implementation of the projects have not been always made, and, if the monitoring did take place (during the project sustainability period), these visits were rather formal. On the other hand, there have been certain deficiencies in the statistical systems of the audited organizations and they could not provide certain information related to PWD, such as the number of people who were able to work among persons with disabilities. Overall, the results have been rather modest due to the fact that, during the implementation and sustainability periods of the projects, no clear requirements have been set out as to the obligation to maintain the number of direct beneficiaries, or any other requirements related to the measurement of the result indicators. Even though the number of direct beneficiaries has not been constant, as set out in the project proposals, no constraints have been established through the secondary legislation in relation to the project executors, in case the number of PWD, as direct beneficiaries, diminished during the project sustainability period.

### Acknowledgements

The authors would like to thank S. Panaite (“Al. I. Cuza” University - Romania) for the technical assistance and I. Macovei (“Al. I. Cuza University” - Romania) for comments and suggestions.
